# Viewing art as a pathway to psychological well‐being and physical health

**DOI:** 10.1111/aphw.70131

**Published:** 2026-02-17

**Authors:** Jennifer E. Stellar, Sascha Priewe, Navdeep K. Lidhar, Loren Martin

**Affiliations:** ^1^ Psychology Department University of Toronto Toronto Canada; ^2^ Aga Khan Museum Toronto Canada

**Keywords:** art, cortisol, emotion, health, heart rate, well‐being

## Abstract

Viewing art in museums is enjoyable and meaningful. Although previous work has found that creating art promotes mental and physical health, whether these benefits extend to passively viewing art is unclear. We manipulated exposure to art by having participants visit a museum exhibit and compared this experience to a neutral and another pleasant activity. To assess physical health, we measured participants' heart rate during these activities and collected salivary cortisol before and after the activity. To assess mental health, participants rated their subjective well‐being and stress. Compared to the neutral or positive activity, viewing art led to greater subjective well‐being and lower stress. Benefits for stress were particularly pronounced for those who began the study with high levels of stress. However, heart rate and cortisol changes did not differ by condition. These results suggest the potential for museum‐based interventions to foster mental health, one hour but were inconclusive concerning physical health.

## INTRODUCTION

Physical and mental health profoundly impact individuals' quality of life. As a result, there has been a proliferation of interventions that aim to enhance both. While many interventions focus on reshaping individuals' perceptions and interpretations of their world (e.g. mindfulness practices and cognitive behavioural therapy; Bolier et al., 2013; Weiss et al., 2016), others aim to modify the physical environment (e.g. spending time in nature, exposure to certain spectrums of indoor lighting; Capaldi et al., 2015; Wang et al., 2024). Museums present a unique and underexplored physical environment with the potential to benefit both mental and physical health (e.g. Chatterjee & Noble, 2016; Fancourt & Finn, 2019). In this preregistered large‐scale study, we examined whether viewing an art exhibit in a museum improved mental and physical health, to test claims that museums can serve as spaces for holistic health promotion.

Art galleries and art exhibitions represent unique, contemplative spaces that provide the opportunity to step outside one's daily routines, offering respite from the stress and distractions of everyday life. This escape into a world of beauty may provide important psychological and physical benefits. Beyond offering a peaceful retreat, museums are also spaces of cultural and historical significance. Increasingly, museums are being reconceptualized as *institutions for human flourishing* (Cotter & Pawelski, 2022), which actively foster physical and mental health (e.g. Chatterjee & Noble, 2016; Cotter et al., 2023). In the past decade, initiatives by health organizations and museums, especially in the United Kingdom, have begun to incorporate free museum visits into traditional treatment strategies for those with physical and mental ailments through social prescription programmes.

Museums have a great deal of potential when considering physical environments as potential interventions for mental and physical health, in part, because they are readily available to many people and widely used. According to UNESCO designations in 2021, there were around 104,000 museums worldwide. Additionally, a nationally representative large‐scale survey (National Endowment for the Arts, 2017) found that around 25% of Americans reported visiting a museum in the past year. Museums may be particularly important spaces for those living in urban or cold climates in which access to natural environments that benefit health and well‐being can be limited. Museums represent a large category that ranges from zoos to science centres. Here, we focus specifically on art exhibits within a museum.

Previous work suggests that engagement with art promotes mental and physical health. Traditionally, this work has focused on active forms of engagement like creating art, which has long been used as a therapeutic tool in clinical settings. Artmaking helps individuals express emotions, process trauma and build self‐esteem (e.g. Joschko et al., 2024; Stuckey & Nobel, 2010). Especially well‐documented is its ability to reduce symptoms of anxiety and depression among both healthy and clinical samples (Jiang et al., 2020; Masika et al., 2020; Newland & Bettencourt, 2020). One particularly rigorous study of artists who were contacted at random intervals 10 times a day, for 1 week (2495 experiences), documented associations between artmaking, flow and positive affect (Holt, 2018). Further, the frequency of artmaking predicted more meaning‐based or eudaimonic forms of happiness.

More recently, research has begun to investigate engagement with art in the form of passive viewing of art created by others, often experts, which is displayed in galleries, art exhibitions or museums. Growing evidence shows that engaging with the arts more generally positively impacts health and well‐being (e.g. Fancourt & Finn, 2019). For instance, a recent meta‐analysis found that receptive cultural engagement was linked to better health and well‐being, though receptive cultural engagement was a broad category that included visits to museums, galleries, art exhibitions, theatres, concerts and cultural festivals (e.g. Viola et al., 2024). In addition, a large Swedish 14‐year cohort study found a lower mortality risk for individuals who most often visited the cinema, concerts, museums and art exhibitions compared to those who rarely did so (Konlaan et al., 2000). While these findings are provocative, they are not specific to viewing art in museums.

More recent work has focused more directly on the benefits of viewing art for mental and physical health. Most of this research targets clinical populations for whom physical or mental health is impaired. For example, vulnerable older adults (65–94) who were referred by health and social care systems participated in 10 weekly sessions in which they visited museums and kept a weekly diary of their experiences (Thomson et al., 2018). From pre‐ to post‐assessment, positive emotions increased, with emotions like enlightened and absorbed increasing most dramatically. A study with adults with dementia and their caregivers found similar effects for measures of quality of life after six visits to a museum for one hour, followed by artmaking activity using a wait‐list control design (Schall et al., 2018). Yet another study of a small group of participants (*N* = 25) with dementia documented decreases in reported depressive symptoms, though no changes in quality of life after a 6‐week program in which they visited a museum and discussed art for two hours (D'Cunha et al., 2019). In this study, salivary cortisol (waking to evening ratio) was higher, but levels of interleukin‐6, a marker of inflammation, did not change from pre‐ to post‐visit assessments.

In healthy adults, one large survey identified a positive association between reports of attending art exhibitions and self‐reported health (Wilkinson et al., 2007). More direct evidence comes from studies where participants attend museums, exhibitions and art galleries. For instance, reports of well‐being increased, and salivary cortisol decreased after visiting a newly restored fresco (Vicoforte Sanctuary; Grossi et al., 2019). Another study with a small sample (*N* = 25) found that cortisol decreased after a brief 45‐min visit to an art gallery, along with reduced self‐reports of stress (Clow & Fredhoi, 2006). Finally, viewing figurative, compared to modern art or a control condition, decreased systolic blood pressure after a brief five minute visit, though no differences in heart rate were found (Mastandrea, Maricchiolo, et al., 2019).

These results led a recent meta‐analysis to suggest there may be important benefits of viewing art on well‐being, though it cautions about the lack of gold‐standard studies with proper control conditions (Trupp et al., 2024). Indeed, the aforementioned studies often have small samples, lack control conditions and sometimes include other activities such as making art or involvement in a larger program, which makes it difficult to interpret their effects. As a result, it is not yet clear whether healthy adults experience benefits simply from viewing art compared to engaging in other activities, especially pleasurable activities. Therefore, we tested whether visiting a museum and attending a visual art exhibition improved mental health and objective markers of physical health compared to a neutral and another pleasant activity in a large sample of healthy young adults.

Why might viewing art be beneficial to health and well‐being? Viewing art is an aesthetic experience, and these experiences are often deeply rewarding (Kawabata & Zeki, 2004), eliciting a variety of positive emotions. Growing work suggests that positive emotions foster subjective well‐being (e.g. Diener et al., 1999) and physical health (e.g. Pressman et al., 2019). Activation of the parasympathetic branch of the autonomic nervous system has been implicated as a mechanistic pathway between momentary experiences of positive emotions and physical health. Positive emotions are associated with greater activation of the vagus nerve, a key component of the parasympathetic system (Oveis et al., 2009; Wang et al., 2013). Vagal activation can directly support physical health through cardiovascular functioning (Kok et al., 2013), predicting lower risk of developing cardiovascular disease (Thayer et al., 2010) and mortality (Jarczok et al., 2022), though these effects are strongly influenced by age, fitness and other health behaviours. It can also indirectly support physical health through its relationship to the HPA‐Axis and inflammatory pathways, where it downregulates cortisol and proinflammatory cytokine responses, respectively (Bonaz et al., 2016; Cuberos Paredes et al., 2025), both of which can be harmful to health when chronically elevated. These biological pathways may explain previous findings linking positive emotions with reduced cortisol and inflammation (Bostock et al., 2011; Joseph et al., 2021; Stellar et al., 2015; Steptoe et al., 2009).

Moreover, art is particularly effective at evoking a positive emotion called awe, which is associated with an appreciation of beauty and the sublime (e.g., Konecni, 2005). In keeping with this claim, recent work has documented that museums and their exhibits, including art exhibits, readily induce feelings of awe along with other positive emotions (Luke, 2021; Luke & Foley, 2025; Price et al., 2019). These studies often find a pronounced and independent effect of awe, which suggests it may play a central role in the benefits of viewing art. There is growing evidence that feeling awe may be especially important for fostering mental and physical health (for a review, see Monroy & Keltner, 2023) and empirical support that it predicts lower levels of proinflammatory cytokines (Stellar et al., 2015) and reduced self‐reported somatic symptoms (Monroy et al., 2023). Further, even acute momentary inductions of awe shift well‐being, stress, and physiological functioning (e.g. Bai et al., 2021; Rudd et al., 2012). Knowing the psychological and potential physical impact of awe, curators of art within museums often consider how to most effectively elicit awe when designing exhibition spaces (Varutti, 2024).

## PRESENT RESEARCH

The present research tested whether visiting a visual art exhibit at a museum would positively impact mental and physical health. To assess mental health, we measured subjective well‐being (Diener et al., 1999), characterized by satisfaction with life, high positive emotion and low negative emotion. We also measured subjective stress. Given the central place of awe within aesthetic experiences, we measured this emotion and awe‐related outcomes that are relevant to well‐being, such as a sense of interconnection and reduced focus on one's own concerns.

To assess physical health, we included two objective measures—heart rate and salivary cortisol. Unfortunately, due to the nature of our recording devices, we were unable to gather more sophisticated features of the heart's rhythm, like heart‐rate variability, which more closely indexes parasympathetic activation. However, previous work suggests that heart‐rate deceleration indicates parasympathetic activation (Berntson et al. 1997; Porges, 2007; Thayer & Lane, 2000), and when chronically elevated, it can be damaging for health (e.g. Ho et al., 2014). We also measured salivary cortisol, a hormonal indicator of stress (Moons et al., 2010), which is also harmful when consistently elevated (e.g., Schoorlemmer et al., 2009).

We hypothesized that visiting a visual art exhibition at a museum would lead to higher subjective well‐being, lower stress, as well as greater awe, less focus on one's own concerns, and a greater sense of interconnection compared to a neutral control condition. We also hypothesized it would impact physical health in the form of heart‐rate deceleration and reduced cortisol from preinduction to post‐induction. We were unsure as to how the art condition would perform compared to a more stringent test, a positive comparison condition that also represented a pleasant activity. We designed this positive comparison condition to be grounded in amusement, rather than awe, allowing us to account for the shared positive valence of the two conditions and more directly assess the unique contributions of viewing art.

This study was preregistered, and all data, syntax, and materials are available on our Open Science Framework site (https://osf.io/c4juu/?view_only=b77d5696fb294d5c9beec0354b98ade1). We note all deviations from our preregistration here. We tested emotions as a dependent measure rather than a mechanism as originally stated and present them as part of the broader construct of subjective well‐being, along with life satisfaction, and negative emotion. We did not analyse creativity or interleukin‐6 due to time constraints that prevented participants from completing the creativity measures and a lack of detectable concentrations of interleukin‐6 due to our salivary collection method. We also did not control for preinduction stress, as it did not differ by condition. All exclusions and other statistical analyses follow our preregistration. This study was approved by the Research Ethics Board at the University of Toronto, Protocol: 33438.

## METHODS

### Participants

We recruited 342 (141 males, 172 females and 29 who declined to state) undergraduates from a large Canadian university in return for course credit. Participants reported most strongly identifying with the following ethnicities: 49.1% East Asia, 22.8% Caucasian, 14.6% South Asian, 8.8% Southeast Asian, 4.7% Middle Eastern, 2.6% African, 1.8% Caribbean and 0.6% South American. A power analysis suggested that a sample size of 246 was required (power = 0.8, *p* = 0.05) to test a small to medium‐sized effect (Cohen's *d* = 0.4) with a between‐subjects ANOVA. We opted to collect additional data to account for missing data and exclusions.

### Procedure

Participants were randomly assigned to one of three conditions (art, neutral and positive) in a between‐subjects design. Participants in the art condition were informed to arrive at the lobby of the Royal Ontario Museum, whereas participants in the neutral and positive comparison conditions were told to come to the laboratory in their university's Psychology Building. Both locations are on the university's campus. Participants arrived in groups ranging from one to seven people. All sessions were held between 10:00 AM and 1:00 PM to minimize the impact of changes in cortisol associated with the circadian rhythm.

Upon arrival, participants provided consent. We collected pre‐nduction salivary cortisol samples using the passive drool technique. Participants were instructed to allow saliva to pool in their mouths without stimulation and then gently expel the accumulated saliva directly into a polypropylene collection tube until they had reached 1 mL, which was marked on the tube, or 5 min had passed. This method was selected to minimize contamination and preserve the integrity of salivary biomarkers.

Participants then completed demographics and individual differences before being fitted with fitness watches (Polar Electro V800) designed to gather heart rate. This device provides a heart‐rate output every second (1‐Hz sampling rate). Once all participants were wearing watches, we gathered pre‐induction heart rate by having participants breathe quietly for two minutes, with their eyes closed, while standing. This duration and method have been successfully used in previous studies to obtain a stable baseline for cardiac measures (Qaiser et al., 2023).

In the art condition, participants were then brought to an immersive visual art exhibit. This exhibit was selected because it presents what the museum curators described as an awe‐inspiring, unique and powerful experience for visitors. The exhibit contained large‐scale installations created out of many intricate pieces of handblown glass. The sculptures are abstract but hint at objects like flowers and are exceptionally colourful. Although this exhibit differs from more traditional visual art exhibits, its complexity, vast scale, and exceptional beauty and intricacy, along with its popularity, as stated by the description of the exhibit by the museum itself, made it an ideal test for the power of encountering expert visual art. Participants were informed not to sit down, not to converse with other participants, and not to use their phones. An experimenter stayed with them in the exhibition to monitor that these rules were being followed. Participants were told to walk around the exhibition at their own pace until the experimenter retrieved them. Participants were free to roam the exhibit for twenty minutes.

In the neutral and positive comparison conditions, participants were given the same instructions while taking part in an activity in a similarly sized space to the art exhibition. These instructions were intended to mimic walking around the art exhibition, though walking pace and spatial movement were not directly measured. Participants were instructed to walk around the basement floor and fill out a paper copy of a blueprint of the floor with the room numbers, which were missing from the document. The room numbers were located on each door. In the neutral condition, participants completed this task without interruption. In the positive comparison condition, participants were interrupted twice while doing this activity to watch two different two‐minute comedic videos played on a computer in the laboratory, which were intended to induce amusement (Gross & Levenson, 1995). Participants stood during the entire activity, even while watching the videos.^i^

The experimenter collected all participants and took them to the lobby of the museum or the laboratory room. Participants then completed surveys, which included measures of their psychological well‐being, stress and emotions (see Section 3.3).^ii^ Saliva was collected a second time using the same method as the preinduction, fifteen minutes after the end of the manipulation, since it takes twenty to forty minutes for cortisol to be detectable in saliva (Kirschbaum & Hellhammer, 1989). Thus, the levels of saliva collected should roughly reflect participants' levels as they were experiencing the twenty‐minute‐long activity. Two minutes before collecting the second saliva sample (13 minutes after the end of the activity), participants were asked to stand quietly while breathing for two minutes to allow for the measurement of their heart rate during a post‐induction period. Participants then returned to their surveys if they had not yet completed them. After completing all surveys, participants were debriefed and dismissed.

### Materials

#### Pre‐induction stress

We measured preinduction levels of perceived stress (*M* = 2.88, *SD* = 0.53) using the Perceived Stress Scale (PSS; Cohen et al., 1983), which assesses how much stress people subjectively experienced over the past month using ten items (*α* = 0.80). Participants reported how often they endorsed statements like, ‘In the past month, how often have you been upset because of something that happened unexpectedly’, from 1 (*Never*) to 5 (*Very Often*). Twenty‐five participants did not complete this scale, leaving a final sample of 317 for this measure.

#### Subjective well‐being

Subjective well‐being is conceptualized as high life satisfaction, high positive affect and low negative affect. We used the Satisfaction with Life Scale (SWLS; Diener et al., 1985), which is an established measure of life satisfaction (*N* = 336; *M* = 4.43, *SD* = 1.09) with five items ranging from 1 (*Strongly disagree*) to 5 (*Strongly agree*). This scale contains items such as ‘So far, I have gotten the important things I want in life’. The scale showed good reliability (*α* = 0.79). Six participants did not complete this item.

Unlike life satisfaction, there are a variety of ways to assess positive and negative affect. We had participants rate how much they felt a variety of negative emotions (*M* = 1.99, *SD* = .78) including: anxious, stressed, bored, sadness, angry (*α* = 0.80) and positive emotions (*M* = 3.04, *SD* = .75) including: happy, calm, excited, interested, inspired and amused (*α* = 0.78) from 1 (*Not at all*) to 5 (*Very much*). This set of emotions comprehensively represented the positive and negative affective space, varying in arousal and pleasantness.

#### Awe and awe‐related measures

In addition, we were interested in awe given its connection to aesthetic experiences (Konecni, 2005). We followed in the tradition of past work (e.g. Stellar et al., 2018), which uses a triplet (awe, amazement and wonder) from the Modified Differential Emotion Scale (Fredrickson, 2013) to measure state experiences of awe. We measured these three emotions with the same anchors as the other positive emotions; the composite showed good reliability (*N* = 340; *α* = 0.71; *M* = 2.75, *SD* = .96). In keeping with our preregistration, we separated awe from our positive emotion measures.^iii^ Three participants did not complete the emotion items.

We asked two additional items related to well‐being, which were not preregistered. These items are typically used in studies of awe, but they are also indicative of positive psychological adjustment. The two items were, ‘In the grand scheme of things, my own issues and concerns felt like they did not matter as much’, (*N* = 338; *M* = 3.92, *SD* = 1.57) and ‘I feel interconnected’, (*N* = 335; *M* = 4.02, *SD* = 1.55) from 1 (*Strongly disagree*) to 7 (*Strongly agree*). Four participants did not complete the former item, and seven did not complete the latter.

#### Post‐induction stress

We used a single‐item measure of stress, *How stressed do you feel about your life at this moment?* (*N* = 328; *M* = 3.11, *SD* = 1.12) on a 5‐point scale from 1 (*Not at all*) to 5 (*Very much*). We used this item instead of the PSS to avoid participants anchoring on their original responses and because the PSS asks about the past month. Fourteen participants did not complete this item.

#### Objective measures of physical health

##### Heart rate (HR)

HR from each participant's mobile watch was exported for each second for both the two‐minute pre‐ and post‐induction periods and the twenty‐minute induction period by two separate research assistants. HR values by second were then aggregated over each 2‐min pre‐ and post‐induction period and the 20‐min induction period. Sixty‐eight participants were missing more than half of the heart‐rate signal from at least one of these periods (pre‐induction, induction or post‐induction), and six more participants had heart‐rate values of more than three SDs from the mean on one of the periods. These participants were removed from heart‐rate analyses as per our preregistration. The final sample was 268 participants in our heart‐rate analyses.

##### Salivary cortisol

We controlled for two variables that index general physical health, body Mass Index (BMI), calculated using participants' own height and weight and weekly hours of exercise. Participants estimated the total number of hours they exercised in the past seven days.^iv^ Following saliva collection, all samples (pre‐ and post) were stored in dry ice in a portable cooler by the experimenters. Following the study, the samples were immediately stored in a −80°C freezer until analysis. Samples were thawed and spun at 2000 rpm at 4°C for 2 min, and cortisol concentrations were determined using Salimetrics High Sensitivity Salivary Cortisol Enzyme Immunoassay kits (State College, PA, Kit#1305503). Samples were assayed using a 1:2 dilution and run in duplicate. Single absorbance readings for samples and standards were obtained at 450 nm using a BioTek Plate reader (Synergy HT, Winooski, VT), and these values were used for the calculation of cortisol levels (ng/mL) based on a linear regression of the standard curve using a log‐logit transformation. The intraassay coefficients of variation ranged from 5.81% to 7.59%, and the interassay coefficient of variation was 8.27%. For the interassay calculation, four replicate points each from high and low cortisol standards were selected. The detection range for the assay was 0.012–3.000 ug/dL.

Forty‐two participants did not provide large enough saliva samples to test cortisol levels at the pre‐ or post‐induction time points, or were missing a label on their sample. For four participants, an error occurred, and the condition was not recorded. Another 62 participants stated they were sick and were removed as per our preregistration. Fourteen more participants did not provide their BMI or hours of exercise. As a result, a total sample of 220 participants was used to measure changes in cortisol from pre‐ to post‐induction.

Although we restricted the time of the study to a 3‐h window to reduce the impact of changes in cortisol due to circadian rhythm (Kirschbaum & Hellhammer, 1989; Matsuda et al., 2012; Mohd Azmi et al., 2021), there were differences in the number of participants collected in each session (see Table 1). Therefore, we deviated from our preregistration and controlled for the time of saliva collection in our analyses related to cortisol (10:00 AM = 1, 11:00 AM = 2 and 12:00 PM = 3). Finally, we log‐transformed cortisol measures at both time points because they were not normally distributed.

**TABLE 1:** Number of participants collected in each condition at each time of day.

Time of session	Art	Positive	Neutral
10:00 AM	22	40	33
11:00 AM	29	31	31
12:00 PM	27	13	8

## RESULTS

### Statistical analysis plan

First, we examined measures of mental health. To create our subjective well‐being measure, we created a composite after *z*‐scoring life satisfaction, positive emotion, and reverse‐scored negative affect. As a result, higher scores represent higher subjective well‐being. We ran an ANOVA for subjective well‐being, but also each measure individually as per our preregistration. Planned contrasts then tested differences between the art and positive comparison condition, as well as the art and neutral condition. Then, we conducted an ANOVA for post‐induction stress and examined whether the art condition compared to the neutral condition was particularly beneficial for high‐stress participants by conducting a regression with preinduction stress, condition (art and neutral) and an interaction between the two.

Finally, we examined measures of physical health. We assessed objective measures, heart rate and cortisol, with a mixed ANOVA that accounted for the within‐subject factor of time (pre‐induction period, induction period and recovery for heart rate and pre‐induction and induction for cortisol), the between‐subjects manipulation of condition and the interaction between the two. These analyses were followed by planned contrasts that compared the art condition to either the positive comparison or neutral condition or changes in time within specific conditions (e.g., changes in cortisol from preinduction to induction period for the art condition). Analyses of salivary cortisol, controlled for BMI, exercise and time of day.

### Mental health

We found an effect of condition on subjective well‐being (see Table 2), such that it was highest in the art condition, followed by the positive comparison and neutral conditions. All conditions were significantly different from each other. Follow‐up ANOVAs revealed an effect of condition for negative and positive emotions,^v^ but not satisfaction with life. Positive emotion was higher in the art than in the neutral and positive comparison conditions. Negative emotion was lower in the art condition compared to the neutral condition, but equivalent to the positive comparison condition.

**TABLE 2:** Means (standard deviations) and significance tests for self‐report variables measured post‐intervention.

Measure	Art condition	Positive comparison	Neutral control	Significance test
Subjective well‐being (z‐scored)	0.28 (.67)^a^	−.001 (.59)^b^	−0.29 (.54)^c^	*F*(2, 332) = 23.47, *p* < .001, *ηp* ^2^ = .12
Satisfaction with life	4.47 (1.11)^a^	4.51 (1.13)^a^	4.29 (1.03)^a^	*F*(2, 329) = 1.26, *p* = .29, *ηp* ^2^ = .008
Positive emotions	3.52 (0.68)^a^	2.86 (0.68)^b^	2.71 (0.64)^b*†*^	*F*(2, 332) = 45.50, *p* < .001, *ηp* ^2^ = .22
Negative emotions	1.89 (0.84)^a^	1.88 (0.72)^a^	2.25 (0.75)^b^	*F*(2, 332) = 7.76, *p <* .001, *ηp* ^2^ = .045
Own concerns less important	4.23 (1.61)^a^	3.66 (1.46)^b^	3.91 (1.60)^b^	*F*(2, 331) = 3.98, *p* = .02, *ηp* ^2^ = .023
Sense of interconnection	4.35 (1.48)^a^	3.76 (1.56)^b^	3.96 (1.59)^a†b^	*F*(2, 328) = 4.16, *p* = .02, *ηp* ^2^ = .025
Post‐induction stress	2.97 (1.12)^a^	3.02 (1.11)^a^	3.34 (1.10)^*b*^	*F*(2, 321) = 3.47, *p* = .03, *ηp* ^2^ = .02

*Note*: Variables with a different superscript are significantly different from one another at *p* < .05. Marginal differences are denoted with a cross.

We also explored individual emotions in more detail (see Table 3). For negative emotions, only stress and anxiety showed significant differences between conditions (see Table 3). Both stress and anxiety were lower in the art condition compared to the neutral, but not the positive comparison condition. For positive emotions, all individual positive emotions were higher in the art condition than in the other two conditions. As expected, the largest effect size was for the emotion of awe. Next, we examined two awe‐related outcomes relevant to well‐being—reduced concern about one's own problems and a sense of interconnection. Condition also impacted both such that they were more strongly endorsed in the art than neutral (though this was marginal for interconnection) and positive comparison conditions.

**TABLE 3:** Means (*SD*), contrasts and omnibus tests for individual emotions.

Emotion	Art condition	Positive condition	Neutral condition	Omnibus test
*Negative emotions*				
Bored	2.28 (1.15)^a^	2.49 (1.08)^ab^	2.75 (1.11)^b *†*^	*F*(2, 332) = 4.71, *p =* .01, *ηp* ^*2*^ = 0.03
Stressed	2.06 (1.19)^a^	1.96 (1.05)^a^	2.61 (1.25)^b^	***F*(2, 332) = 9.85, *p <* .001, *ηp*** ^***2***^ **= 0.06**
Anxious	1.88 (1.00)^a^	1.90 (0.99)^a^	2.42 (1.18)^b^	***F*(2, 330) = 9.12, *p <* .001, *ηp*** ^***2***^ **= 0.05**
Sad	1.71 (0.99)^ab^	1.56 (0.86)^b^	1.85 (0.94)^a^	*F*(2, 332) = 2.71, *p* = .068, *ηp* ^*2*^ = 0.016
Angry	1.53 (0.97)^a^	1.50 (0.92)^a^	1.58 (0.87)^a^	*F*(2, 331) = 0.18, *p* = .84, *ηp* ^*2*^ = 0.001
*Positive emotions*				
Calm	3.89 (0.95)^a^	3.63 (.95)^b^	3.29 (1.04)^c^	***F*(2, 330) = 10.27, *p* < .001, *ηp*** ^***2***^ **= 0.06**
Interest	3.78 (1.00)^a^	3.08 (1.04)^b^	2.93 (1.08)^b^	***F*(2, 332) = 20.98, *p* < .001, *ηp*** ^***2***^ **= 0.11**
Happy	3.63 (0.91)^a^	3.16 (1.03)^b^	2.82 (0.92)^c^	***F*(2, 332) = 19.72, *p* < .001, *ηp*** ^***2***^ **= 0.11**
Awe	3.43 (0.89)^a^	2.39 (0.77)^b^	2.43 (0.83)^b^	***F*(2, 333) = 57.49, *p* < .001, *ηp*** ^**2**^ **= .26**
Amused	3.42 (1.07)^a^	2.92 (1.24)^b^	2.65 (1.03)^b *†*^	***F*(2, 331) = 13.17, *p* < .001, *ηp*** ^***2***^ **= 0.07**
Inspiration	3.37 (1.15)^a^	2.06 (0.96)^b^	2.17 (1.07)^b^	***F*(2, 332) = 53.46, *p* < .001, *ηp*** ^***2***^ **= 0.24**
Excited	3.03 (1.11)^a^	2.35 (0.97)^b^	2.41 (0.93)^b^	***F*(2, 332) = 15.49, *p* < .001, *ηp*** ^***2***^ **= 0.09**

*Note*: When individual emotions are significantly different from each other, they are denoted by a different superscript letter. All significant omnibus values after Bonferroni correction (*p* ≤ .003) are bolded.

**FIGURE 1:**
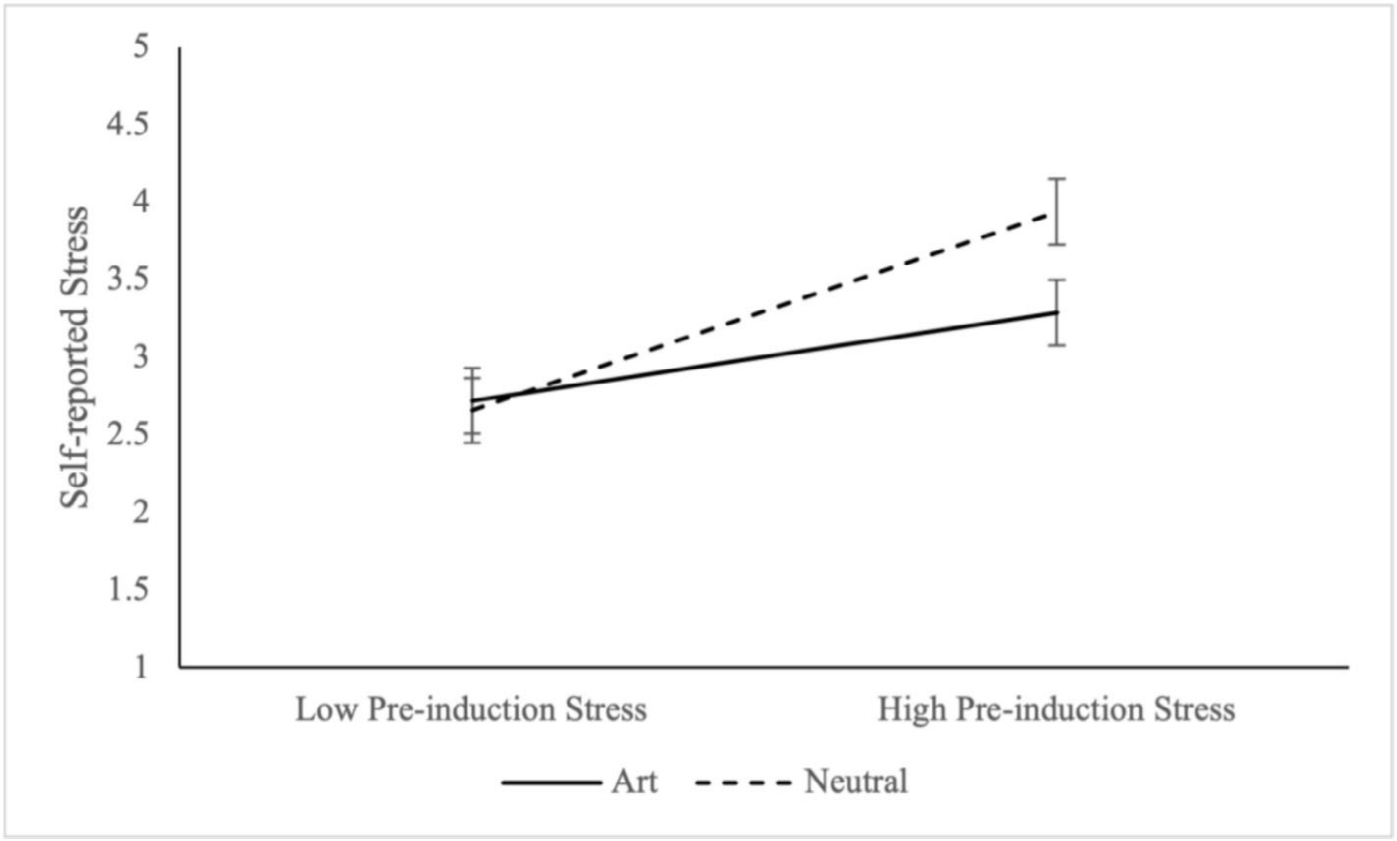
Mean (standard error) for current stress in the art and neutral conditions for those with high and low preinduction stress levels.

Finally, condition impacted reports of post‐induction stress, such that post‐induction stress was lower in the art compared to the neutral condition, but equivalent to the positive comparison condition (See Table 2). We also conducted an exploratory analysis to identify whether preinduction stress moderated the effect of condition on self‐reported current stress after the induction. Specifically, we tested whether the art induction was more powerful for those who were more stressed compared to less stressed to begin with. We ran a regression with the condition (neutral = 0, art = 1), preinduction stress and the interaction between the two predicting current stress. When comparing the art to neutral condition, we found a main effect of pre‐induction stress, *B* = 1.27, *SE* = .21, *t* = 6.16, *p* < .001, 95% CI: .86, 1.68, an effect of condition, *B* = .1.74, *SE* = .86, *t* = 2.02, *p* = .04, 95% CI: .04, 3.45 and an interaction between the two, *B* = −.70, *SE* = .29, *t* = 2.40, *p* = .02, 95% CI: −1.28, −.13 (see Figure 1).

The simple effects revealed that when comparing the art and neutral conditions, the art condition led to significantly lower levels of stress for those with higher levels (+1 *SD*) of pre‐induction stress, *B* = −.65, *SE* = .21, *t* = 3.16, *p* = .002, 95% CI: −1.05, −.24, but not for those with lower levels (−1 *SD*) of pre‐induction stress, *B* = .06, *SE* = .21, *t* = .26, *p* = .79, 95% CI: −.36, .47. These results suggest that the art induction had a more powerful effect on current stress for those with greater levels of preinduction stress.

### Physical health

**FIGURE 2:**
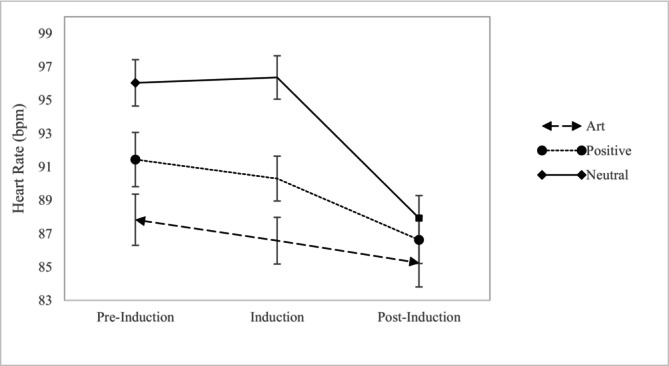
Mean (standard error) heart rate at preinduction, induction and post‐induction periods for each condition.

We conducted a mixed ANOVA with condition (art, positive and neutral) as a between‐subjects factor, time (preinduction, induction and post‐induction) as a within‐subjects variable and the interaction between the two. We found a main effect of condition, *F*(2, 263) = 6.89, *p* = .001, *ηp* ^2^ = .05, time, *F*(2, 526) = 42.11, *p* < .001, *ηp* ^2^ = .14, and an interaction between the two, *F*(4, 526) = 6.15 and *p* < .001, *ηp* ^2^ = .05 in predicting heart rate (see Figure 2).^vi^

Looking within conditions, participants in the art condition experienced a marginal decline in heart rate from pre‐induction to induction, *t*(88) = 1.81, *p* = .07, *d* = .19, whereas participants in the positive comparison, *t*(94) = 1.03, *p* = .15, *d* = .11, and neutral, *t*(81) = −0.37, *p* = .73, *d* = .04, conditions did not. Participants in the positive comparison, *t*(94) = 3.46, *p* < .001 and *d* = .36 and neutral, *t*(81) = 6.44, *p* < .001, *d* = .71, conditions showed significant declines in heart rate from induction to post‐induction, but not participants in the art condition, *t*(88) = 1.56, *p* = .12, *d* = .17. These results suggest that while the induction marginally decreased heart rate in the art condition from pre‐induction levels, it did not for the control conditions, which exhibited declines from the induction to the post‐induction period.

Further examining the main effect of condition on heart rate revealed differences at pre‐induction, *F*(2, 263) = 6.98, *p* < .001, *ηp* ^2^ = .05, and during the induction, *F*(2, 263) = 12.83, *p* < .001, *ηp* ^2^ = .09, but not post‐induction, *F*(2, 263) = 0.88, *p* = .41, *ηp* ^2^ = .007. At pre‐induction and during the induction, participants in the art condition had significantly lower heart rate than the neutral condition (Preinduction: *Contrast Estimate*: 8.21, 95% CI: 3.88, 12.54, *p* < .001; Induction: *Contrast Estimate*: 9.78, 95% CI: 5.95, 13.61, *p* < .001). They also had lower heart rate in the art than the positive comparison condition (Preinduction: *Contrast Estimate*: 3.61, 95% CI: −.57, 7.78, *p* = .09, Induction: *Contrast Estimate*: 3.72, 95% CI: 0.31, 7.41, *p* = .03), though this effect was marginal for the pre‐induction.

Further examination of the interaction effect revealed only two significant interactions. The first occurred when comparing the neutral to the art condition from induction to post‐induction, *F*(1,167) = 20.34, *p* < .001, *ηp* ^2^ = .11. The second occurred when comparing the positive comparison condition to the art condition from the induction to post‐induction, *F*(1,181) = 3.36, *p* = .07, *ηp* ^2^ = .02. Specifically, there was a greater decrease in heart rate from the induction to post‐induction for the neutral condition and marginally more for the positive comparison condition than the art condition. There were no interactions from pre‐induction to induction (art vs neutral: *F*(1,169) = 1.88, *p* = .17, *ηp* ^2^ = .01 and art versus positive emotion: *F*(1,181) = 0.02, *p* = .90, *ηp* ^2^ = .00).

**FIGURE 3:**
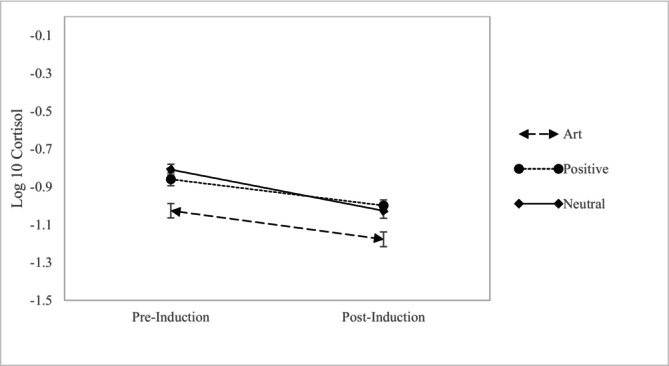
Mean (standard error) log‐transformed cortisol levels at pre‐ and post‐induction for each condition.

For salivary cortisol, we conducted a repeated measures ANOVA with condition (art, positive and neutral) as a between‐subjects factor, time (preinduction and post‐induction) as a within‐subjects variable and the interaction between the two, controlling for BMI, amount of exercise and the time at which the saliva collection occurred (see Figure 3). There was a main effect of condition, *F*(2, 214) = 5.90, *p* = .003, *ηp* ^2^ = .05, but no effect of time, *F*(1, 214) = .11, *p* = .74, *η* ^2^ = .001, nor an interaction between the two, *F*(2, 214) = 1.73, *p* = .18, *ηp* ^2^ = .016.^vii^

Further examination of the main effect of condition revealed differences in cortisol by condition were present at both pre‐induction, *F*(2, 214) = 6.38, *p* = .002, *η* ^2^ = .056 and post‐induction, *F*(2, 214) = 3.97, *p* = .02, *η* ^2^ = .036. Specifically, pre‐induction and post‐induction cortisol were significantly lower in the art condition than the neutral (Pre‐induction: *Contrast Estimate*: .16, 95% CI: .07, .26, *p* < .001, Post‐induction: *Contrast Estimate*: .10, 95% CI: −.001, .20, *p* = .051) and positive comparison (Pre‐induction: *Contrast Estimate*: .12, 95% CI: .03, .22, *p* = .008; Post‐induction: *Contrast Estimate*: .14, 95% CI: .04, .24, *p* = .006) conditions.

## DISCUSSION

Although our work cannot speak to the impact of museums broadly, we find powerful effects of viewing art at a museum on mental health. In our study, attending an art exhibit led to higher subjective well‐being. Interestingly, these effects were driven by the affective side of subjective well‐being as life satisfaction was not significantly different across conditions, though this may reflect that life satisfaction is designed to be a trait‐measure and therefore may be less susceptible to contextual changes. Viewing art in a museum was associated with greater positive emotions compared to the neutral and positive comparison conditions. This was true for each positive emotion we measured, though the largest effect occurred for the emotion of awe. This finding is in keeping with awe's strong ties to aesthetics and beauty (e.g., Konecni, 2005). In addition, awe‐related outcomes such as feeling one's own concerns were less important, and a sense of interconnection was higher in the art condition than the other two conditions, though marginally for interconnection compared to the positive comparison condition.

Viewing art in a museum also led to lower negative emotions compared to the neutral, but not positive, comparison condition, specifically less stress and anxiety. This finding was corroborated by our additional self‐report of perceived stress about one's own issues. Although stress was measured with a single item, which carries limitations, it builds confidence that we found similar effects for the stress and anxiety items within the larger set of negative emotions we collected.

Interestingly, viewing art in a museum had a stronger impact on momentary stress for individuals who were more stressed to begin with, compared to less stressed. These results suggest more pronounced benefits for high‐stress samples or during times when stress is particularly high. Overall, these findings for mental health were consistent with past work that has found benefits of art interventions (Schall et al., 2018; Thomson et al., 2018) and for claims that museums can be institutions that support flourishing (Cotter & Pawelski, 2022).

In terms of physical health, the results were inconclusive. Heart rate effects were generally not very robust, though there were marginal decreases in heart rate when viewing art in the museum, but not in the positive comparison and neutral conditions, from pre‐induction to induction. Moreover, these effects need to be considered in the context of the pre‐induction period, which has notable differences that suggest greater caution in interpreting these results. We found that participants already had lower heart rate at the pre‐induction period in the art condition compared to the neutral condition and marginally lower than the positive comparison condition. This may reflect that heart rate was collected in the museum lobby in the art condition versus the psychology laboratory in the other two conditions. Second, heart rate declined more strongly from induction to post‐induction in the positive comparison and neutral conditions. This may reflect that heart rate was higher in those two conditions to begin with, allowing more room for deceleration. Further, we were unable to calculate heart rate variability due to the restrictions of the watch, which would have provided a superior cardiovascular measure.

In addition, we did not find any evidence that cortisol changed across time based on condition. Again, unexpectedly, preinduction cortisol levels were already significantly lower in art compared to the other two conditions. Although we cannot be sure, these differences may reflect the benefits of anticipating a visit to a museum in the case of cortisol, since preinduction collection happened when only this knowledge was accessible to participants. Future work should consider whether simply anticipating a visit to a museum may offer benefits to psychological and mental health. The findings for physical health were less consistent with previously documented physical health improvements from encountering art (Clow & Fredhoi, 2006; Grossi et al., 2019; Mastandrea, Fagioli, & Biasi, 2019; Wilkinson et al., 2007). Overall, our results support broader claims that museums have the potential to foster flourishing (Cotter & Pawelski, 2022), when it comes to promoting mental health.

Since art exhibits in museums are accessible to the public, they may offer important spaces to foster health and well‐being. However, claims about the importance of viewing art in museums must come from participant samples that are large and diverse and include rigorous controls to consider the impact of viewing art in a museum compared to other potentially less time‐intensive and costly activities. Further, as viewing art in museums is incorporated into social prescription programs, which aim to improve mental and physical health, objective markers of physical health should be incorporated into self‐report measures to ensure the efficacy of museums in impacting objective markers of physical health. This work offers one step in this direction. It suggests potential benefits to positive affect that were not seen in the positive comparison condition, in addition to reduced stress and negative affect that occurred to a similar degree as the positive comparison condition. The physical health benefits were not as clear, tempering strong claims about museum‐based interventions' impact on physical health.

Based on our findings, viewing art in a museum was most impactful on stress levels for those with high levels of stress. It may be important to identify periods in which stress is high or groups of individuals who are prone to stress to test whether viewing art at a museum holds even stronger benefits in these contexts. If so, then identifying those who could benefit most would be the first step in ensuring that the benefits of an art intervention are maximized.

Although we aimed to improve upon the methods used in previous studies of viewing art, there are some notable limitations to our study. First, one critical methodological limitation is that we had participants meet us in two different locations, depending on the condition: the museum or the psychology laboratory. Ideally, we would have collected our pre‐induction assessments in the same location to remove their impact on pre‐induction measures. As a result, our pre‐induction measures were affected and incorporated anticipation of either seeing an exhibit or partaking in a more traditional psychology experiment with respect to cortisol, as well as being in these different environments with respect to heart rate. While using repeated measures analysis does reduce the impact of these differences, they are still present and add caution to interpreting these findings.

Another methodological limitation concerns the effectiveness of our positive comparison condition. Even the focal emotion in the positive comparison condition, amusement, was lower than in the art condition, though the positive emotion comparison was higher for happiness, calm and marginally for amusement than the neutral condition. A more appropriate interpretation of this condition is that it represented a pleasant or positive activity with which to compare the art condition, but it was not one that fully controlled for the intensity of positive emotions.

Second, there were some important limitations to our measures. Our mobile collection devices could not assess interbeat interval, which would have allowed us to calculate heart rate variability, a superior measure of stress. With improvements in mobile devices in the past five years, future work could gather more sophisticated measures of autonomic nervous system activity out in the field. We also did not measure participants' spatial movement or walking. If there are differences in movement across conditions, it would impact measures of heart rate. In addition, it is worth noting that participants' mean levels of heart rate, while in the acceptable range, were on the higher side. It may stem from the increased stress and cognitive focus that comes with participating in an experiment in a novel setting. The mean heart rate for this sample was also higher during the pre‐induction than in the induction period, which may seem counterintuitive since participants could move around in the induction period. However, the space they were able to walk in was small enough that it was likely that participants were standing still the majority of the time, and the calming effect of the art condition may have outweighed increases in heart rate from movement.

In addition, we only measured well‐being through subjective well‐being (Diener et al., 1999). Although this is a common method for measuring well‐being, there are also measures of well‐being that are more tailored for use in museums, such as the Museum Well‐being Measure Toolkit (Thomson & Chatterjee, 2015), which we regrettably did not include. Many of the items are shared, and this toolkit includes additional items related to the social experiences of art, which we did not wish to capture, but their inclusion would have offered a more well‐rounded and contextually grounded assessment of well‐being. Further, we only measured stress with a single item. Future work would be better served by using an established state measure of stress that included more items, which would have included more items.

Third, there are limits to the generalizability of our findings. Our sample of undergraduates is less representative of the typical museum goers, and future work should replicate these findings with an older community sample. In addition, our study focused specifically on the benefits of seeing art in a museum. We urge caution in extending these findings to other types of museums, since this is a broad category that ranges from zoos to science museums. Further, we used a specific exhibition for our intervention. To increase the generalizability of these findings, future work should include multiple different types of exhibitions or even different types of art. Past work suggests figurative art is particularly moving for individuals and may have stronger effects (Mastandrea, Maricchiolo, et al., 2019). Finally, the controlled setting of our art exhibit did not allow people to visit the exhibit with others or for as much time as they may have wanted, which can introduce measurement confounds and reduce ecological validity. Museums can foster well‐being through many pathways, including as social spaces that strengthen social connection, which we did not allow for here.

## CONCLUSION

Physical spaces like museums that introduce beauty through visual art are important shared spaces to foster mental health. They may be designed to showcase an artist's creativity and expose us to new perspectives and ideas, but they also offer spaces for humans to flourish.

## CONFLICT OF INTEREST STATEMENT

The authors declare no conflicts of interest.

## ETHICS STATEMENT

I certify that we have complied with the APA ethical principles regarding research with human participants in the conduct of the research presented in this manuscript. Our ethics protocol was approved by the University of Toronto (Protocol number: 33438). The authors have no conflicting or competing interests to declare.

## Supporting information


**Data S1.** Supporting Information.

## Data Availability

The data, syntax and measures collected as part of this research are available on Open Science Framework: https://osf.io/c4juu/?view_only=b77d5696fb294d5c9beec0354b98ade1.

## ENDNOTES

i We had to switch the comedy sketches halfway through the experiment due to the content of the videos, but controlling for this change did not impact the results in any way.

ii We also collected interpersonal measures, which we did not preregister here because they are part of a preregistration for a separate project.

iii We also asked participants to endorse appraisals relating to awe (vastness and the need for accommodation), which we do not analyse here.

iv We deviated from our preregistration and did not covary out ethnicity, since this covariate is relevant for inflammation measures, which we preregistered, but were unable to gather in this study.

v In line with our preregistration we did not include the awe composite in our measure of positive emotions, but the effect of condition was stronger when awe is included, *F*(2, 333) = 48.52, *p* < .001, *ηp* ^2^ = .23.

vi We preregistered that we would also run our analyses for the first, second and third 5‐min segments. These analyses show the same pattern of results as the full induction and are in the supplement for interested readers.

vii We maximized available data by including participants who stated they were sick and without covariates (since some participants did not complete the covariates) and found the same pattern: Only a significant main effect of time, *F*(1, 292) = 20.14, *p* < .001, *η* ^2^ = .065, in which cortisol decreased across the experiment.
