# Supplementary material for: Viewing art as a pathway to psychological well‐being and physical health
Source: Appl Psychol Health Well Being. 2026 Feb 17;18(1):e70131. doi: 10.1111/aphw.70131 (PMC12910538; doi:10.1111/aphw.70131)
Supplement: Supplementary file 1 — Data S1. Supporting Information. [file APHW-18-0-s001.docx]

**Additional Heart rate Analyses**

In considering changes in heart rate across time, we found a main effect of condition, F(2, 263)=6.59, *p*=.002, ηp^2^ =.05, time, F(2, 526)=44.04, *p*<.001, ηp^2^ =.14, and an interaction between the two, F(4, 526)=5.90, *p*=.003, ηp^2^ =.04 in predicting heart rate for the first five minutes. In considering changes in heart rate across time, we found a main effect of condition, F(2, 263)=7.36, *p*<.001, ηp^2^ =.05, time, F(2, 526)=34.67, *p*<.001, ηp^2^ =.12, and an interaction between the two, F(4, 526)=6.29, *p*=.002, ηp^2^ =.05 in predicting heart rate for the second five minutes. In considering changes in heart rate across time, we found a main effect of condition, F(2, 263)=6.58, *p*=.002, ηp^2^ =.05, time, F(2, 526)=35.93, *p*<.001, ηp^2^ =.12, and an interaction between the two, F(4, 526)=5.49, *p*=.005, ηp^2^ =.04 in predicting heart rate for the last five minutes. These patterns are the same as using the entire induction period, which appears in the main text.
